# Bis[1-cyclo­propyl-6-fluoro-4-oxo-7-(1-piperazin-4-ium-1-yl)-1,4-dihydro­quinoline-3-carboxyl­ate-κ^2^ *O* ^3^,*O* ^4^]bis­(nitrato-κ*O*)copper(II)

**DOI:** 10.1107/S1600536812007830

**Published:** 2012-02-29

**Authors:** Juan Yang, Shi-Wei Yan, Zhong-Li Ye, Guang-Hua Xin, Suo-Cheng Chang

**Affiliations:** a College of Chemistry and Chemical Engineering, Southwest University, Chongqing 400715, People’s Republic of China

## Abstract

In the title complex, [Cu(NO_3_)_2_(C_17_H_18_FN_3_O_3_)_2_], the Cu^II^ ion is located on an inversion center. It exhibits a distorted octa­hedral geometry, being coordinated by six O atoms, four from two ciprofloxacin ligand mol­ecules (*L*), which act as bidentate ligands, and two from two nitrate anions. In the ligand, the piperazine ring has a chair conformation and the quinoline system is essentially planar [maximum deviation = 0.097 (2) Å]. One of the nitrate O atoms is disordered over two positions [occupancy ratio = 0.51 (6):0.49 (6)]. There is a C—H⋯F inter­action in the complex. In the crystal, mol­ecules are linked *via* N—H⋯O hydrogen bonds generating a two-dimensional network lying parallel to (111). The presence of C—H⋯O inter­actions leads to the formation of a three-dimensional structure. The title complex was prepared by hydro­thermal synthesis, and the hexa­hydrate form of this complex, synthesized by conventional methods, has been reported previously [Hernandez-Gil *et al.* (2009 ▶). *Polyhedron*, **28**, 138–144].

## Related literature

For general background on the use of quinolones in the treatment of infections, see: Barbas *et al.* (2006 ▶); Basavoju *et al.* (2006 ▶); Xiao *et al.* (2005 ▶). For the synthesis and crystal structure of the hexa­hydrate form of this complex, see: Hernandez-Gil *et al.* (2009 ▶).
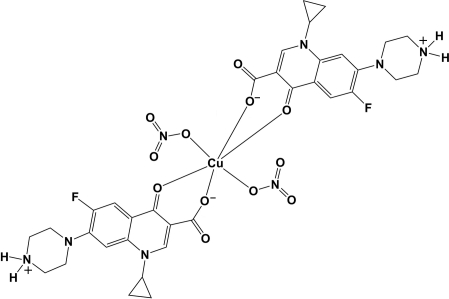


## Experimental

### 

#### Crystal data

[Cu(NO_3_)_2_(C_17_H_18_FN_3_O_3_)_2_]
*M* *_r_* = 850.25
Triclinic, 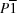

*a* = 8.8921 (18) Å
*b* = 9.863 (2) Å
*c* = 11.186 (2) Å
α = 77.62 (3)°
β = 81.95 (3)°
γ = 64.15 (3)°
*V* = 861.1 (3) Å^3^
*Z* = 1
Mo *K*α radiation
μ = 0.73 mm^−1^
*T* = 293 K
0.50 × 0.48 × 0.35 mm

#### Data collection

Bruker APEX CCD area-detector diffractometer
Absorption correction: multi-scan (*SADABS*; Sheldrick, 1996 ▶) *T*_min_ = 0.713, *T*_max_ = 0.785
4702 measured reflections
2935 independent reflections
2766 reflections with *I* > 2σ(*I*)
*R*_int_ = 0.026

#### Refinement

*R*[*F* ^2^ > 2σ(*F* ^2^)] = 0.039
*wR*(*F* ^2^) = 0.136
*S* = 1.00
2935 reflections
269 parameters
H-atom parameters constrained
Δρ_max_ = 0.43 e Å^−3^
Δρ_min_ = −0.34 e Å^−3^

### 

Data collection: *SMART* (Bruker, 2001 ▶); cell refinement: *SAINT* (Bruker, 2001 ▶); data reduction: *SAINT*; program(s) used to solve structure: *SHELXS97* (Sheldrick, 2008 ▶); program(s) used to refine structure: *SHELXL97* (Sheldrick, 2008 ▶); molecular graphics: *SHELXTL-Plus* (Sheldrick, 2008 ▶); software used to prepare material for publication: *SHELXL97*.

## Supplementary Material

Crystal structure: contains datablock(s) I, global. DOI: 10.1107/S1600536812007830/su2380sup1.cif

Structure factors: contains datablock(s) I. DOI: 10.1107/S1600536812007830/su2380Isup2.hkl

Additional supplementary materials:  crystallographic information; 3D view; checkCIF report

## Figures and Tables

**Table 1:** Hydrogen-bond geometry (Å, °)

*D*—H⋯*A*	*D*—H	H⋯*A*	*D*⋯*A*	*D*—H⋯*A*
N3—H3*A*⋯O2^i^	0.90	1.86	2.749 (3)	170
N3—H3*B*⋯O4^ii^	0.90	2.00	2.838 (19)	155
N3—H3*B*⋯O6^ii^	0.90	2.21	2.995 (3)	146
C13—H13*A*⋯O4^iii^	0.97	2.40	3.25 (3)	147
C13—H13*B*⋯O5^i^	0.97	2.58	3.382 (3)	140
C15—H15*A*⋯O3^iv^	0.97	2.57	3.514 (3)	165
C17—H17*A*⋯F1	0.97	2.18	2.857 (3)	125

Symmetry codes: (i) 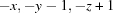; (ii) 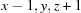; (iii) 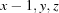; (iv) 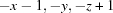.

**Fig. 1.**
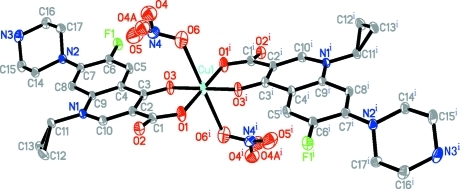
The molecular structure of the title compound, with the atom numbering and displacement ellipsoids drawn at the 30% probability level. H atoms have been omitted for clarity [Symmetry code: (i) -x, -y, -z].

## supplementary crystallographic information

### Comment

Ciprofloxacin is member of a class of quinolones used to treat infections (Barbas *et al.*, 2006; Basavoju *et al.*, 2006; Xiao *et al.* 2005). The title copper(II) complex was prepared by mixing Ciprofloxacin [cyclopropyl-6-fluoro-1,4-dihydro-4-oxo-7-(1-pip -erazinyl)-3-quinolinecarboxylic acid, L] with Cu(NO_3_)_2_ under hydrothermal conditions. The synthesis and crystal structure of the hexahydrate form of this complex have been described by (Hernandez-Gil *et al.*, 2009). Herein, we report on the crystal structure of the title complex.

The asymmetric unit of the title compound is compossed of one Cu^II^ ion, that is located on an inversion center, one L ligand and one NO_3_^-^ anion (Fig. 1). The Cu^II^ ion is coordinated by six O atoms, four from two *L* ligand molecules and two from two NO_3_^-^ anions, in a distorted octahedral geometry. There is a C—H···F interaction in the complex. In the ligand the piperazine ring (N2,N3,C14—C17) has a chair conformation and the quinoline moiety (N1,C2—C10) is essentially planer [max. devsion = 0.097 (2) Å].

In the crystal, molecules are linked *via* N—H···O hydrogen bonds generating a two-dimensional network lieing parallel to (1 1 1). The presence of C—H···O interactions leads to the formation of a three-dimensional structure.

The geometrical parameters of the title compound are very similar to those of the hexhydrate form of this complex (Hernandez-Gil *et al.*, 2009).

### Experimental

A mixture of Cu(NO_3_)_2_^.^3H_2_O (0.121 g, 0.5 mmol), cyclopropyl-6-fluoro-1,4-dihydro-4-oxo-7-(1-pip -erazinyl)-3-quinolinecarboxylic acid (HL; 0.192 g, 0.5 mmol) in distilled water (7 ml), was stirred for 20 min in air. The mixture was then transferred to a 23 ml Teflon-lined hydrothermal bomb. The bomb was kept at 383 K for 72 h under autogenous pressure. Upon cooling, blue block-like crystals of the title compound were obtained from the reaction mixture.

### Refinement

The NH H atoms were located in a difference Fourier map. In the final cycles of refinement all the H atoms were included in calculated positions and refined as riding atoms: N—H = 0.90 Å, C—H = 0.97 Å, with *U*_iso_(H) = 1.2 *U*_eq_(N,*C*). One of the nitrate O atoms (O4) is disordered over two positions [occupancy ratio 0.51 (6):0.49 (6)].

### Crystal data

**Table:**

[Cu(NO_3_)_2_(C_17_H_18_FN_3_O_3_)_2_]	*Z* = 1
*M**_r_* = 850.25	*F*(000) = 439
Triclinic, *P*1	*D*_x_ = 1.640 Mg m^−^^3^
Hall symbol: -P 1	Mo *K*α radiation, λ = 0.71073 Å
*a* = 8.8921 (18) Å	Cell parameters from 4702 reflections
*b* = 9.863 (2) Å	θ = 1.9–25.0°
*c* = 11.186 (2) Å	µ = 0.73 mm^−^^1^
α = 77.62 (3)°	*T* = 293 K
β = 81.95 (3)°	Block, blue
γ = 64.15 (3)°	0.50 × 0.48 × 0.35 mm
*V* = 861.1 (3) Å^3^	

### Data collection

**Table:**

Bruker APEX CCD area-detector diffractometer	2935 independent reflections
Radiation source: fine-focus sealed tube	2766 reflections with *I* > 2σ(*I*)
Graphite monochromator	*R*_int_ = 0.026
phi and ω scans	θ_max_ = 25.0°, θ_min_ = 1.9°
Absorption correction: multi-scan (*SADABS*; Sheldrick, 1996)	*h* = −10→10
*T*_min_ = 0.713, *T*_max_ = 0.785	*k* = −11→11
4702 measured reflections	*l* = −13→13

### Refinement

**Table:**

Refinement on *F*^2^	Primary atom site location: structure-invariant direct methods
Least-squares matrix: full	Secondary atom site location: difference Fourier map
*R*[*F*^2^ > 2σ(*F*^2^)] = 0.039	Hydrogen site location: inferred from neighbouring sites
*wR*(*F*^2^) = 0.136	H-atom parameters constrained
*S* = 1.00	*w* = 1/[σ^2^(*F*_o_^2^) + (0.124*P*)^2^] where *P* = (*F*_o_^2^ + 2*F*_c_^2^)/3
2935 reflections	(Δ/σ)_max_ = 0.010
269 parameters	Δρ_max_ = 0.43 e Å^−^^3^
0 restraints	Δρ_min_ = −0.34 e Å^−^^3^

### Special details

**Table:**

Geometry. Bond distances, angles *etc*. have been calculated using the rounded fractional coordinates. All su's are estimated from the variances of the (full) variance-covariance matrix. The cell e.s.d.'s are taken into account in the estimation of distances, angles and torsion angles
Refinement. Refinement of *F*^2^ against ALL reflections. The weighted *R*-factor *wR* and goodness of fit *S* are based on *F*^2^, conventional *R*-factors *R* are based on *F*, with *F* set to zero for negative *F*^2^. The threshold expression of *F*^2^ > σ(*F*^2^) is used only for calculating *R*-factors(gt) *etc*. and is not relevant to the choice of reflections for refinement. *R*-factors based on *F*^2^ are statistically about twice as large as those based on *F*, and *R*- factors based on ALL data will be even larger.

### Fractional atomic coordinates and isotropic or equivalent isotropic displacement parameters (Å^2^)

**Table:**

	*x*	*y*	*z*	*U*_iso_*/*U*_eq_	Occ. (<1)
Cu1	0.00000	0.00000	0.00000	0.0353 (2)	
F1	−0.35238 (18)	0.05781 (15)	0.56641 (12)	0.0421 (4)	
O1	0.0564 (2)	−0.19893 (19)	−0.03746 (15)	0.0468 (5)	
O2	0.13735 (19)	−0.44723 (18)	0.00732 (14)	0.0393 (5)	
O3	−0.12571 (19)	−0.03396 (17)	0.14953 (13)	0.0363 (5)	
O4	0.471 (2)	−0.314 (4)	0.2079 (17)	0.121 (5)	0.51 (6)
O5	0.2223 (3)	−0.2423 (4)	0.2922 (2)	0.1016 (12)	
O6	0.2794 (3)	−0.1278 (3)	0.1173 (2)	0.0720 (8)	
N1	−0.14125 (19)	−0.43566 (19)	0.32933 (15)	0.0255 (5)	
N2	−0.3720 (2)	−0.2050 (2)	0.69774 (16)	0.0292 (5)	
N3	−0.4207 (3)	−0.2834 (2)	0.95656 (17)	0.0443 (6)	
N4	0.3188 (3)	−0.2350 (3)	0.20525 (19)	0.0497 (8)	
C1	0.0559 (2)	−0.3170 (2)	0.03294 (18)	0.0302 (6)	
C2	−0.0471 (2)	−0.3005 (2)	0.15213 (18)	0.0267 (6)	
C3	−0.1197 (2)	−0.1630 (2)	0.20359 (18)	0.0276 (6)	
C4	−0.1884 (2)	−0.1755 (2)	0.32806 (17)	0.0264 (6)	
C5	−0.2417 (3)	−0.0520 (2)	0.39143 (19)	0.0315 (6)	
C6	−0.2984 (3)	−0.0651 (2)	0.51032 (19)	0.0304 (6)	
C7	−0.3090 (2)	−0.2001 (2)	0.57654 (18)	0.0271 (6)	
C8	−0.2596 (2)	−0.3220 (2)	0.51328 (17)	0.0267 (5)	
C9	−0.1994 (2)	−0.3108 (2)	0.39076 (17)	0.0237 (5)	
C10	−0.0668 (2)	−0.4268 (2)	0.21705 (17)	0.0271 (6)	
C11	−0.1454 (2)	−0.5794 (2)	0.39316 (18)	0.0283 (6)	
C12	−0.1739 (3)	−0.6783 (3)	0.3222 (2)	0.0432 (8)	
C13	−0.3071 (3)	−0.5953 (3)	0.4123 (2)	0.0376 (7)	
C14	−0.4089 (3)	−0.3377 (3)	0.75118 (19)	0.0328 (7)	
C15	−0.5121 (3)	−0.3117 (3)	0.8697 (2)	0.0410 (7)	
C16	−0.3878 (3)	−0.1462 (3)	0.9029 (2)	0.0420 (7)	
C17	−0.2872 (3)	−0.1690 (3)	0.78328 (19)	0.0347 (6)	
O4A	0.449 (3)	−0.3490 (11)	0.1929 (13)	0.089 (5)	0.49 (6)
H3B	−0.48200	−0.26860	1.02760	0.0530*	
H5A	−0.23800	0.03950	0.35130	0.0380*	
H3A	−0.32310	−0.36560	0.97240	0.0530*	
H10A	−0.02510	−0.51320	0.18030	0.0330*	
H11A	−0.06810	−0.63430	0.45940	0.0340*	
H12A	−0.11320	−0.78830	0.34430	0.0520*	
H12B	−0.19280	−0.64120	0.23560	0.0520*	
H13A	−0.40620	−0.50830	0.37990	0.0450*	
H13B	−0.32660	−0.65540	0.48860	0.0450*	
H14A	−0.30510	−0.42850	0.76610	0.0390*	
H14B	−0.46950	−0.35400	0.69400	0.0390*	
H15A	−0.61880	−0.22440	0.85430	0.0490*	
H15B	−0.53360	−0.40080	0.90530	0.0490*	
H16A	−0.32700	−0.12930	0.95960	0.0500*	
H16B	−0.49310	−0.05670	0.88960	0.0500*	
H17A	−0.27240	−0.07680	0.74670	0.0420*	
H17B	−0.17730	−0.25190	0.79800	0.0420*	
H8A	−0.26680	−0.41200	0.55330	0.0320*	

### Atomic displacement parameters (Å^2^)

**Table:**

	*U*^11^	*U*^22^	*U*^33^	*U*^12^	*U*^13^	*U*^23^
Cu1	0.0494 (3)	0.0258 (3)	0.0272 (3)	−0.0183 (2)	0.0170 (2)	−0.0044 (2)
F1	0.0648 (8)	0.0285 (7)	0.0343 (7)	−0.0209 (6)	0.0109 (6)	−0.0136 (5)
O1	0.0742 (11)	0.0322 (9)	0.0315 (8)	−0.0263 (9)	0.0212 (8)	−0.0074 (7)
O2	0.0513 (9)	0.0299 (8)	0.0326 (8)	−0.0148 (7)	0.0116 (7)	−0.0113 (6)
O3	0.0487 (8)	0.0213 (8)	0.0304 (8)	−0.0131 (7)	0.0152 (6)	−0.0018 (6)
O4	0.064 (5)	0.163 (13)	0.055 (4)	0.015 (7)	0.000 (3)	0.005 (8)
O5	0.0879 (17)	0.178 (3)	0.0495 (13)	−0.0775 (19)	0.0127 (12)	−0.0038 (16)
O6	0.0717 (13)	0.0698 (15)	0.0623 (13)	−0.0261 (12)	0.0013 (11)	0.0021 (11)
N1	0.0326 (8)	0.0233 (9)	0.0226 (8)	−0.0148 (7)	0.0013 (6)	−0.0028 (6)
N2	0.0388 (9)	0.0288 (9)	0.0221 (8)	−0.0178 (8)	0.0051 (7)	−0.0053 (7)
N3	0.0597 (12)	0.0404 (11)	0.0237 (9)	−0.0153 (10)	0.0072 (8)	−0.0059 (8)
N4	0.0666 (14)	0.0604 (16)	0.0317 (11)	−0.0352 (13)	−0.0028 (10)	−0.0082 (10)
C1	0.0362 (10)	0.0317 (11)	0.0246 (10)	−0.0165 (9)	0.0020 (8)	−0.0059 (8)
C2	0.0314 (9)	0.0259 (10)	0.0228 (10)	−0.0133 (8)	0.0009 (7)	−0.0029 (7)
C3	0.0300 (9)	0.0243 (10)	0.0260 (10)	−0.0110 (8)	0.0014 (8)	−0.0018 (8)
C4	0.0304 (9)	0.0223 (10)	0.0250 (10)	−0.0116 (8)	0.0017 (8)	−0.0019 (8)
C5	0.0417 (10)	0.0237 (10)	0.0302 (10)	−0.0177 (9)	0.0027 (8)	−0.0008 (8)
C6	0.0389 (10)	0.0258 (11)	0.0279 (10)	−0.0148 (9)	0.0029 (8)	−0.0077 (8)
C7	0.0286 (9)	0.0301 (11)	0.0237 (10)	−0.0137 (9)	0.0020 (7)	−0.0059 (8)
C8	0.0327 (9)	0.0270 (10)	0.0220 (9)	−0.0160 (9)	−0.0004 (7)	−0.0004 (7)
C9	0.0268 (9)	0.0224 (10)	0.0228 (9)	−0.0119 (8)	−0.0009 (7)	−0.0028 (7)
C10	0.0311 (9)	0.0258 (10)	0.0247 (10)	−0.0123 (8)	0.0022 (8)	−0.0064 (8)
C11	0.0353 (10)	0.0239 (11)	0.0279 (10)	−0.0156 (9)	0.0010 (8)	−0.0038 (8)
C12	0.0637 (14)	0.0417 (13)	0.0394 (12)	−0.0367 (12)	0.0117 (11)	−0.0148 (10)
C13	0.0439 (12)	0.0423 (13)	0.0343 (11)	−0.0290 (11)	0.0060 (9)	−0.0033 (9)
C14	0.0410 (11)	0.0338 (12)	0.0267 (11)	−0.0208 (10)	0.0035 (8)	−0.0036 (9)
C15	0.0488 (12)	0.0411 (13)	0.0314 (11)	−0.0231 (11)	0.0090 (10)	−0.0006 (9)
C16	0.0591 (13)	0.0348 (12)	0.0294 (11)	−0.0160 (11)	0.0017 (10)	−0.0107 (9)
C17	0.0457 (11)	0.0331 (11)	0.0279 (10)	−0.0177 (10)	0.0016 (9)	−0.0101 (8)
O4A	0.132 (11)	0.040 (7)	0.050 (5)	−0.006 (4)	0.011 (5)	0.004 (3)

### Geometric parameters (Å, º)

**Table:**

Cu1—O1	1.9267 (18)	C4—C5	1.408 (3)
Cu1—O3	1.9293 (16)	C4—C9	1.406 (3)
Cu1—O6	2.637 (3)	C5—C6	1.355 (3)
Cu1—O1^i^	1.9267 (18)	C6—C7	1.413 (3)
Cu1—O3^i^	1.9293 (16)	C7—C8	1.399 (3)
Cu1—O6^i^	2.637 (3)	C8—C9	1.399 (3)
F1—C6	1.352 (2)	C11—C13	1.495 (4)
O1—C1	1.260 (3)	C11—C12	1.500 (3)
O2—C1	1.243 (2)	C12—C13	1.504 (4)
O3—C3	1.268 (2)	C14—C15	1.505 (3)
O4—N4	1.23 (3)	C16—C17	1.503 (3)
O4A—N4	1.229 (17)	C5—H5A	0.9300
O5—N4	1.216 (4)	C8—H8A	0.9300
O6—N4	1.239 (3)	C10—H10A	0.9300
N1—C9	1.402 (3)	C11—H11A	0.9800
N1—C11	1.458 (3)	C12—H12A	0.9700
N1—C10	1.339 (3)	C12—H12B	0.9700
N2—C7	1.392 (3)	C13—H13A	0.9700
N2—C14	1.470 (3)	C13—H13B	0.9700
N2—C17	1.480 (3)	C14—H14A	0.9700
N3—C15	1.489 (4)	C14—H14B	0.9700
N3—C16	1.492 (3)	C15—H15A	0.9700
N3—H3A	0.9000	C15—H15B	0.9700
N3—H3B	0.9000	C16—H16A	0.9700
C1—C2	1.505 (3)	C16—H16B	0.9700
C2—C10	1.370 (3)	C17—H17A	0.9700
C2—C3	1.433 (3)	C17—H17B	0.9700
C3—C4	1.443 (3)		
			
O1—Cu1—O3	93.27 (8)	C7—C8—C9	121.13 (17)
O1—Cu1—O6	86.89 (9)	N1—C9—C4	118.31 (17)
O1—Cu1—O1^i^	180.00	C4—C9—C8	120.46 (17)
O1—Cu1—O3^i^	86.74 (8)	N1—C9—C8	121.16 (17)
O1—Cu1—O6^i^	93.11 (9)	N1—C10—C2	125.14 (18)
O3—Cu1—O6	90.88 (8)	N1—C11—C13	119.88 (18)
O1^i^—Cu1—O3	86.74 (8)	N1—C11—C12	119.26 (17)
O3—Cu1—O3^i^	180.00	C12—C11—C13	60.31 (16)
O3—Cu1—O6^i^	89.12 (8)	C11—C12—C13	59.67 (17)
O1^i^—Cu1—O6	93.11 (9)	C11—C13—C12	60.02 (17)
O3^i^—Cu1—O6	89.12 (8)	N2—C14—C15	110.3 (2)
O6—Cu1—O6^i^	180.00	N3—C15—C14	109.6 (2)
O1^i^—Cu1—O3^i^	93.27 (8)	N3—C16—C17	110.1 (2)
O1^i^—Cu1—O6^i^	86.89 (9)	N2—C17—C16	110.8 (2)
O3^i^—Cu1—O6^i^	90.88 (8)	C4—C5—H5A	120.00
Cu1—O1—C1	129.10 (15)	C6—C5—H5A	120.00
Cu1—O3—C3	125.15 (14)	C7—C8—H8A	119.00
Cu1—O6—N4	127.7 (2)	C9—C8—H8A	119.00
C9—N1—C10	119.56 (17)	N1—C10—H10A	117.00
C9—N1—C11	119.69 (16)	C2—C10—H10A	117.00
C10—N1—C11	120.40 (16)	N1—C11—H11A	115.00
C7—N2—C14	115.73 (17)	C12—C11—H11A	115.00
C7—N2—C17	116.41 (19)	C13—C11—H11A	115.00
C14—N2—C17	112.28 (18)	C11—C12—H12A	118.00
C15—N3—C16	110.14 (18)	C11—C12—H12B	118.00
O4—N4—O5	122.7 (10)	C13—C12—H12A	118.00
O4—N4—O6	113.2 (11)	C13—C12—H12B	118.00
O5—N4—O6	122.6 (3)	H12A—C12—H12B	115.00
O4A—N4—O5	118.8 (8)	C11—C13—H13A	118.00
O4A—N4—O6	117.4 (7)	C11—C13—H13B	118.00
C15—N3—H3B	110.00	C12—C13—H13A	118.00
C16—N3—H3A	110.00	C12—C13—H13B	118.00
C15—N3—H3A	110.00	H13A—C13—H13B	115.00
H3A—N3—H3B	108.00	N2—C14—H14A	110.00
C16—N3—H3B	110.00	N2—C14—H14B	110.00
O1—C1—C2	119.11 (17)	C15—C14—H14A	110.00
O1—C1—O2	122.29 (19)	C15—C14—H14B	110.00
O2—C1—C2	118.60 (17)	H14A—C14—H14B	108.00
C3—C2—C10	118.39 (18)	N3—C15—H15A	110.00
C1—C2—C3	123.98 (17)	N3—C15—H15B	110.00
C1—C2—C10	117.58 (17)	C14—C15—H15A	110.00
C2—C3—C4	116.32 (17)	C14—C15—H15B	110.00
O3—C3—C2	125.45 (18)	H15A—C15—H15B	108.00
O3—C3—C4	118.23 (17)	N3—C16—H16A	110.00
C3—C4—C9	121.71 (17)	N3—C16—H16B	110.00
C3—C4—C5	120.11 (18)	C17—C16—H16A	110.00
C5—C4—C9	118.15 (18)	C17—C16—H16B	110.00
C4—C5—C6	120.65 (19)	H16A—C16—H16B	108.00
F1—C6—C5	118.54 (18)	N2—C17—H17A	110.00
C5—C6—C7	122.64 (18)	N2—C17—H17B	110.00
F1—C6—C7	118.78 (18)	C16—C17—H17A	109.00
N2—C7—C8	123.21 (18)	C16—C17—H17B	110.00
N2—C7—C6	119.81 (17)	H17A—C17—H17B	108.00
C6—C7—C8	116.94 (18)		
			
O3—Cu1—O1—C1	−20.0 (2)	C16—N3—C15—C14	59.7 (3)
O6—Cu1—O1—C1	70.7 (2)	C15—N3—C16—C17	−58.6 (3)
O3^i^—Cu1—O1—C1	160.0 (2)	O1—C1—C2—C3	−12.9 (3)
O6^i^—Cu1—O1—C1	−109.3 (2)	O1—C1—C2—C10	169.7 (2)
O1—Cu1—O3—C3	15.06 (19)	O2—C1—C2—C3	167.3 (2)
O6—Cu1—O3—C3	−71.87 (18)	O2—C1—C2—C10	−10.1 (3)
O1^i^—Cu1—O3—C3	−164.94 (19)	C1—C2—C3—O3	9.7 (3)
O6^i^—Cu1—O3—C3	108.13 (18)	C1—C2—C3—C4	−169.18 (18)
O1—Cu1—O6—N4	−59.5 (2)	C10—C2—C3—O3	−172.9 (2)
O3—Cu1—O6—N4	33.7 (2)	C10—C2—C3—C4	8.3 (3)
O1^i^—Cu1—O6—N4	120.5 (2)	C1—C2—C10—N1	173.62 (19)
O3^i^—Cu1—O6—N4	−146.3 (2)	C3—C2—C10—N1	−4.0 (3)
Cu1—O1—C1—O2	−159.28 (17)	O3—C3—C4—C5	−7.5 (3)
Cu1—O1—C1—C2	20.9 (3)	O3—C3—C4—C9	174.55 (19)
Cu1—O3—C3—C2	−13.6 (3)	C2—C3—C4—C5	171.5 (2)
Cu1—O3—C3—C4	165.31 (14)	C2—C3—C4—C9	−6.5 (3)
Cu1—O6—N4—O4	149.8 (15)	C3—C4—C5—C6	−176.6 (2)
Cu1—O6—N4—O5	−44.3 (4)	C9—C4—C5—C6	1.4 (4)
C10—N1—C9—C4	4.7 (3)	C3—C4—C9—N1	0.1 (3)
C10—N1—C9—C8	−172.32 (19)	C3—C4—C9—C8	177.10 (19)
C11—N1—C9—C4	177.84 (18)	C5—C4—C9—N1	−177.9 (2)
C11—N1—C9—C8	0.9 (3)	C5—C4—C9—C8	−0.9 (3)
C9—N1—C10—C2	−2.8 (3)	C4—C5—C6—F1	−178.3 (2)
C11—N1—C10—C2	−175.92 (19)	C4—C5—C6—C7	−0.6 (4)
C9—N1—C11—C12	147.5 (2)	F1—C6—C7—N2	−0.9 (3)
C9—N1—C11—C13	76.9 (2)	F1—C6—C7—C8	176.9 (2)
C10—N1—C11—C12	−39.4 (3)	C5—C6—C7—N2	−178.5 (2)
C10—N1—C11—C13	−110.0 (2)	C5—C6—C7—C8	−0.7 (4)
C14—N2—C7—C6	168.5 (2)	N2—C7—C8—C9	178.99 (19)
C14—N2—C7—C8	−9.2 (3)	C6—C7—C8—C9	1.3 (3)
C17—N2—C7—C6	−56.3 (3)	C7—C8—C9—N1	176.45 (19)
C17—N2—C7—C8	126.0 (2)	C7—C8—C9—C4	−0.5 (3)
C7—N2—C14—C15	−166.8 (2)	N1—C11—C12—C13	−109.8 (2)
C17—N2—C14—C15	56.3 (3)	N1—C11—C13—C12	108.7 (2)
C7—N2—C17—C16	168.18 (19)	N2—C14—C15—N3	−58.1 (3)
C14—N2—C17—C16	−55.2 (3)	N3—C16—C17—N2	55.6 (3)

Symmetry code: (i) −*x*, −*y*, −*z*.

### Hydrogen-bond geometry (Å, º)

**Table:**

*D*—H···*A*	*D*—H	H···*A*	*D*···*A*	*D*—H···*A*
N3—H3*A*···O2^ii^	0.90	1.86	2.749 (3)	170
N3—H3*B*···O4^iii^	0.90	2.00	2.838 (19)	155
N3—H3*B*···O6^iii^	0.90	2.21	2.995 (3)	146
C13—H13*A*···O4^iv^	0.97	2.40	3.25 (3)	147
C13—H13*B*···O5^ii^	0.97	2.58	3.382 (3)	140
C15—H15*A*···O3^v^	0.97	2.57	3.514 (3)	165
C17—H17*A*···F1	0.97	2.18	2.857 (3)	125

Symmetry codes: (ii) −*x*, −*y*−1, −*z*+1; (iii) *x*−1, *y*, *z*+1; (iv) *x*−1, *y*, *z*; (v) −*x*−1, −*y*, −*z*+1.
